# Type I interferons augment regulatory T cell polarization in concert with ancillary cytokine signals

**DOI:** 10.3389/frtra.2023.1149334

**Published:** 2023-04-17

**Authors:** Siawosh K. Eskandari, Hazim Allos, Jenelle M. Safadi, Ina Sulkaj, Jan S. F. Sanders, Paolo Cravedi, Irene M. Ghobrial, Stefan P. Berger, Jamil R. Azzi

**Affiliations:** ^1^ Transplantation Research Center, Division of Nephrology, Brigham and Women's Hospital, Harvard Medical School, Boston, MA, United States; ^2^ Division of Nephrology, University Medical Center Groningen, University of Groningen, Groningen, Netherlands; ^3^ Perelman School of Medicine, University of Pennsylvania, Philadelphia, PA, United States; ^4^ Graduate Program in Immunology, Johns Hopkins School of Medicine, Baltimore, MD, United States; ^5^ Translational Transplant Research Center, Division of Nephrology, Icahn School of Medicine at Mount Sinai, New York City, NY, United States; ^6^ Department of Medical Oncology, Dana-Farber Cancer Institute, Harvard Medical School, Boston, MA, United States

**Keywords:** type interferons, interferon alpha, interferon beta, interleukin, foxp regulatory t cells, treg induction

## Abstract

In the transplant community, research efforts exploring endogenous alternatives to inducing tolerogenic allo-specific immune responses are much needed. In this regard, CD4 ^+ ^FoxP3^+^ regulatory T cells (T_regs_) are appealing candidates due to their intrinsic natural immunosuppressive qualities. To date, various homeostatic factors that dictate T_reg_ survival and fitness have been elucidated, particularly the non-redundant roles of antigenic CD3*ζ*/T-cell-receptor, co-stimulatory CD28, and cytokine interleukin (IL-)2 dependent signaling. Many of the additional biological signals that affect T_regs_ remain to be elucidated, however, especially in the transplant context. Previously, we demonstrated an unexpected link between type I interferons (IFNs) and T_regs_ in models of multiple myeloma (MM)—where MM plasmacytes escaped immunological surveillance by enhancing type I IFN signaling and precipitating upregulated T_reg_ responses that could be overturned with specific knockdown of type I IFN signaling. Here, we elaborated on these findings by assessing the role of type I IFN signaling (IFN-α and -β) on T_reg_ homeostasis within an alloimmune context. Specifically, we studied the induction of T_regs_ from naïve CD4 T cells. Using *in vitro* and *in vivo* models of murine skin allotransplantation, we found that type I IFN indeed spatiotemporally enhanced the polarization of naïve CD4 T cells into FoxP3^+^ T_regs_. Notably, however, this effect was not independent of, and rather co-dependent on, ancillary cytokine signals including IL-2. These findings provide evidence for the relevance of type I IFN pathway in modulating FoxP3^+^ T_reg_ responses and, by extension, stipulate an additional means of facilitating T_reg_ fitness *via* type I IFNs.

## GRAPHICAL ABSTRACT

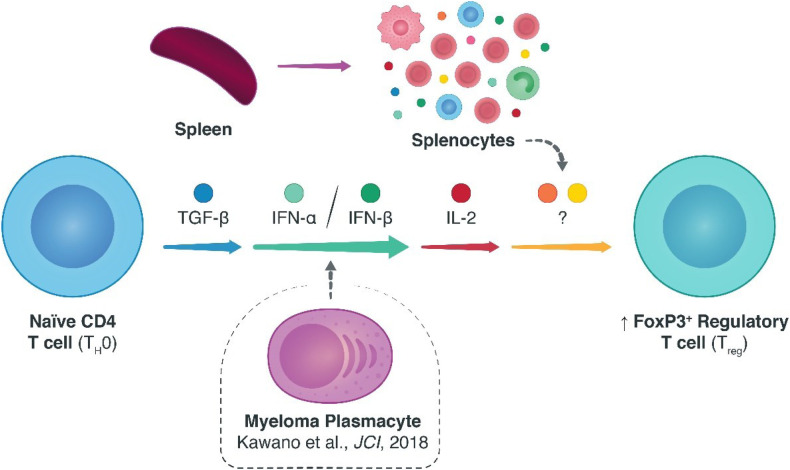

## Introduction

Interferons (IFNs) are a prototypical class of cytokines that were discovered in 1950s because of their aptitude in interfering with viral replication (1). A growing corpus of evidence, however, has since implicated IFNs in markedly more cellular processes than viral interference alone, including anti-tumor immunity, angiogenesis, and immunomodulation of both the innate and adaptive arms of immunity (2–4). Unsurprisingly, perturbations in IFN signaling luxate a multitude of oncological, autoimmune, and viral diseases (5). Herein, the IFN signaling pathways can be clustered in three subtypes—I through III—each mode of signaling recruiting distinct surface-tethered interferon receptors (1). The type I IFN family, in particular, consists of subtypes α, β, δ, ε, κ, τ, and ω, among which the α and β IFNs are the most broadly expressed and IFN-β displays the strongest affinity for its physiological receptor, the type I IFN receptor (IFNAR) (3, 6).

While the effector roles of type I IFNs have been studied in varying tumor and auto-immune niches, their clinical potential in prolonging allograft survival remains poorly defined. Previously, our group studied the role of type I IFNs in animal models of multiple myeloma, finding that myeloma cells in the tumor microenvironment recruit FoxP3^+^ regulatory T cells (T_regs_) through IFNAR signaling to escape immune surveillance (4). Notably, depleting FoxP3^+^ T_regs_ in myeloma-inoculated mice reinstated immunological recognition of the oncogenic cells and facilitated tumor remission—with T_reg_-specific ablation of IFNAR recapitulating this very effect. In addition, a recent article by Fueyo-González and colleagues in *Immunity* showed that IFN-β has an immunomodulatory role in allo-immunity through CD4^+^-specific IFNAR recruitment by enhancing T_reg_ induction and stabilizing the FoxP3^+^ T_reg_ phenotype—particularly, utilizing phosphorylated signal transducer and activator of transcription (pSTAT)1 and p300 to acetylate FoxP3 (7, 8). In a T_reg_-dependent, major histocompatibility complex (MHC-) mismatched heart transplant model, IFN-β treatment was able to synergize with cytotoxic T-lymphocyte-associated protein 4 immunoglobulin (CTLA-4-Ig) therapy to decidedly lengthen cardiac allograft survival—although, IFN-β treatment on its own could not prolong allograft survival (7).

Building on the mounting evidence in the field linking type I IFNs and T_reg_ homeostasis, we forayed into studying the impact of type I IFN signaling on FoxP3^+^ T_reg_ induction in the context of allo-immune pathology using *in vitro* T_reg_ induction assays and an *in vivo* model of allogeneic skin transplantation.

## Methods

### Study design

This study was designed to investigate the impact of type I IFN signaling on the induction of CD4 ^+ ^FoxP3^+^ T_regs_ from naïve CD4^+^ helper T cells in the context of allotransplantation. To this end, experiments were designed wherein naïve CD4^+^ T cells were steered towards an induced regulatory T cell (T_reg_) phenotype in transforming growth factor beta 1 (TGF-β1-)mediated T_reg_ induction assays, in the presence and absence of IL-2 and with varying dosages of IFN-α and IFN-β. The aim of these experiments was to assess the phenotypic stability and activation state of the induced CD4 ^+ ^FoxP3^+^ T_regs_ and the conventional CD4 ^+ ^FoxP3^−^ T cells (T_cons_), *via* flow cytometric staining of FoxP3 and Ki-67 respectively (Supplementary Figure S1A). In addition to these *in vitro* experiments, we set out to ascertain the effects of type I IFNs on *in vivo* CD4 ^+ ^FoxP3^+^ induction using Rag1^−/−^ mice transplanted with fully MHC-mismatched skin grafts. Specifically, we focused on the phenotype and activation state of the CD4 ^+^ Foxp3^+^ T_regs_ and CD4 ^+ ^FoxP3^−^ T_cons_ using flow cytometry on the splenic and graft-draining lymphoid cells of the transplanted mice (Supplementary Figure S1B). The animals were randomly assigned to treatment groups and age-matched between conditions where necessary. The investigators were not blinded, sample sizes were determined based on prior experiences, and no animals were excluded because of illness.

### Materials

Murine anti-CD3ɛ (145-2C11, #16-0031-85) and murine anti-CD28 (37.51, #16-0281-85) were obtained from Invitrogen. Recombinant human IL-2 (#200-02-250 ug) with cross-species reactivity to murine cells was obtained from PeproTech, and recombinant mouse IFN-alpha 2 (#12100-1) and IFN-beta (#8234-MB) were purchased from R&D Systems.

Full culture medium was prepared by supplementing RPMI 1640 (#10-040-CV, Corning) with 10% BenchMark Fetal Bovine Serum (#100–106, Gemini), 1% penicillin/streptomycin (#30-002-CI, Corning), 1 × GlutaMAX (#35050061, Gibco), 25 mM HEPES (#15630080, Gibco), and 55 μM β-mercaptoethanol (#21985023, Gibco). Dulbecco's 1 × PBS in all experiments was from Corning (#21-031-CV, Corning). For all *in vitro* assays, Corning 96-well tissue-culture-treated flat-bottom microplates (#CLS3595, Sigma) were used unless otherwise specified. All remaining chemicals and solvents were purchased from Millipore Sigma, and all reagents were used as received unless otherwise noted.

### Mice

*C57BL/6* (#000664), *BALB/c* (#000651), and *B6.129S7-Rag1^tm1Mom^* (Rag1^−/−^, #002216), mice were purchased from The Jackson Laboratories. All murine strains in this study were maintained in specific-pathogen-free (SPF) conditions at the Brigham and Women's Hospital animal facility in accordance with federal, state, and institutional guidelines. The study protocol was approved by the Brigham and Women's Hospital Institutional Animal Care and Use Committee (IACUC). Mice were sex- and age-matched (8–12 weeks old).

### In vitro assays

Magnetic-activated cell sorting (MACS-)sorted CD4 ^+ ^CD25^−^ T cells were divided into non-stimulated (NS), NS plus plate-bound anti-CD3 (1 μg/ml), plate-bound anti-CD28 (1 μg/ml), and soluble TGF-β1 (10 ng/ml; TGF-β1), TGF-β1 plus soluble IL-2 (20 ng/ml; +IL-2), TGF-β1 plus soluble IFN-α_low_ or IFN-β_low_ (1,000 IU/ml; +IFN-α_low_ or + IFN-β_low_), or TGF-β1 plus soluble IFN-α_high_ or IFN-β_high_ (10,000 IU/ml; +IFN-α_high_ or + IFN-β_high_) conditions. Per condition, per well, 5 × 10^4^ cells were cultured at 37°C and 5% CO_2_ in 200 μl volumes of full culture medium (see “Materials” subheading above) in flat-bottom microplates for three days. This TGF-β-dependent FoxP3^+^ T_reg_ induction experiment was adapted from the protocol described by Fantini and colleagues (9).

For the experiments using splenocytes, TGF-β1, +IFN-α_low_, +IFN-α_high_, +IFN-β_low_, and + IFN-β_high_ conditions were cultured for three days starting with 5 × 10^4^ cells. The splenocytes were incubated at 37°C and 5% CO_2_ in 200 μl volumes of full culture medium in flat-bottom microplates with increasing concentrations of IL-2 in the range of 0–200 ng/ml.

Throughout the culture period, well supernatants were not replenished with full culture medium or other adjuvants, and per condition, per concentration three technical replicates were plated.

### Adoptive cell transfer

1.0 × 10^6^ CD3ɛ^+^CD25^−^ T cells were injected retro-orbitally in 90 μl total volumes of 1 × DPBS at day three post-transplant. To test the *in vivo* efficacy of type I IFNs in augmenting FoxP3^+^ T_reg_ induction, three treatment conditions were tested: (1) PBS, (2) IFN-α, and (3) IFN-β. On days zero through six, the PBS-treated mice received once-daily intraperitoneal injections of PBS while the IFN-α- and IFN-β-treated mice received daily injections of 10,000 IU IFN-α and IFN-β resuspended in PBS respectively. Animals were euthanized at day seven post adoptive transfer for mechanistic studies.

### Statistics

Differences between two normally distributed groups were analyzed with independent samples two-tailed Student *t*-tests, and non-parametric Mann-Whitney *U*-tests when the assumption of homoscedasticity could not be met. Statistical analyses of multiple groups were performed with one-way analyses of variance followed by Holm-Šídák multiple comparison tests, or mixed-effects model analyses with the Geisser-Greenhouse correction followed by Tukey multiple comparison tests for experimental groups with matched data points across multiple time points or concentrations. *P* < 0.05 was considered significant for all analyses. Data analysis and graphing were performed with Prism 9.3.1 (GraphPad Software). Graphs show boxplots with median, interquartile range, minimum, maximum, and all individual data points of the denoted experimental groups.

## Results

### In vitro type I IFN signaling augments FoxP3 T_reg_ induction in naïve CD4 T cells

**Figure 1:**
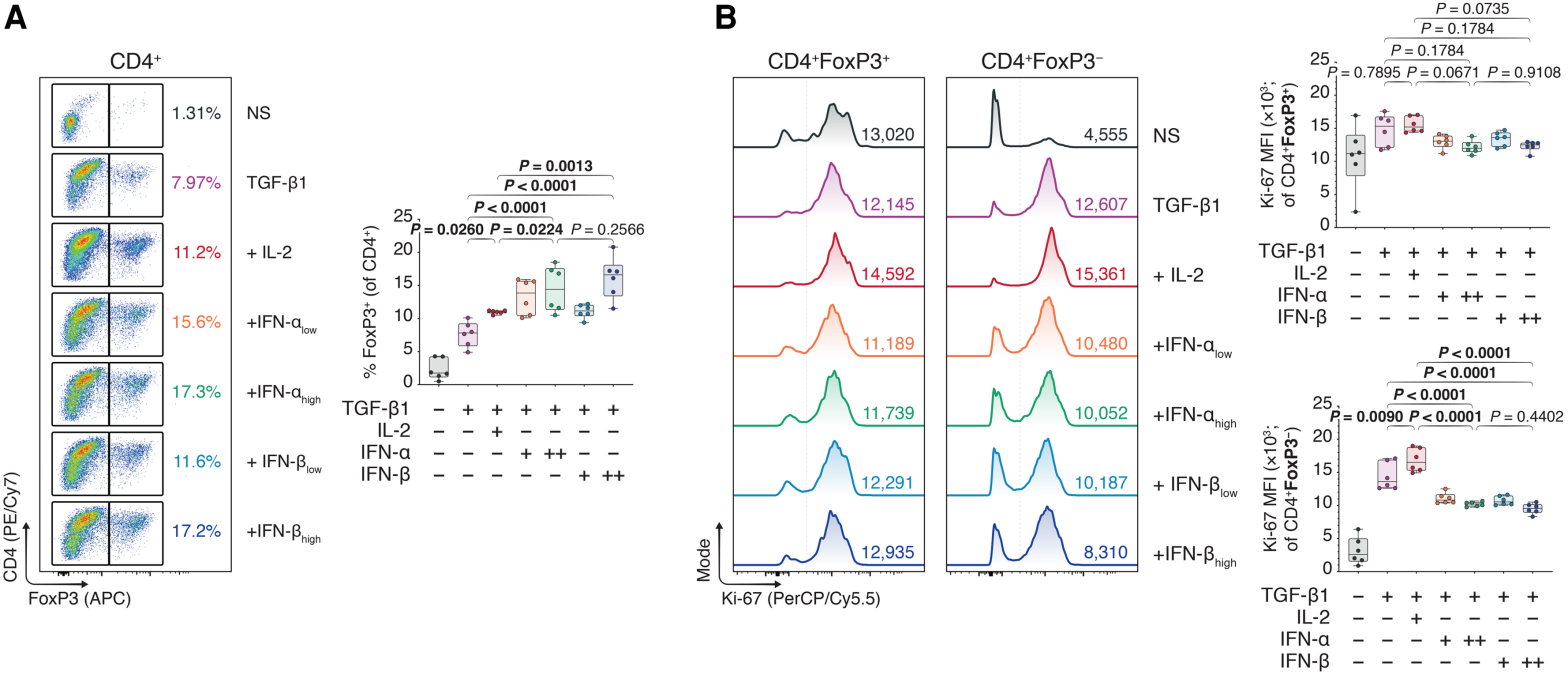
*Type I interferons augment Foxp3 induction in naïve, conventional helper CD4 T cells.* Murine CD4 ^+ ^CD25^−^ T cells were magnetically isolated as per the manufacturer's instructions and expanded for three days without stimulants (NS), or with anti-CD3 (1 μg/ml), anti-CD28 (1 μg/ml), and TGF-β1 (10 ng/ml; TGF-β1). In separate wells, IL-2 (20 ng/ml; +IL-2), IFN-α_low_ (1,000 IU/ml; +IFN-α_low_), IFN-α_high_ (10,000 IU/ml; IFN-α_high_), IFN-β_low_ (1,000 IU/ml; +IFN-β_low_), or IFN-β_high_ (10,000 IU/ml; IFN-β_high_) were added to the TGF-β1 condition (*n* = 3 technical replicates/condition; 2 experiments). (**A**) Flow cytometric analysis and box plots of FoxP3^+^ T_regs_ among CD4^+^ T cells. (**B**) Flow cytometric analysis and box plots of Ki-67 mean fluorescence intensities (MFIs) among CD4 ^+ ^FoxP3^+^ T_regs_ and CD4 ^+ ^FoxP3^−^ non-T_regs_. Data represent boxplots with median, interquartile range, minimum, maximum, and all individual data points of the denoted experimental groups. *P* values were calculated with one-way analyses of variance followed by Holm-Šídák multiple comparison tests were performed. IFN-α, interferon alpha; IFN-β, interferon beta; IL-2, interleukin 2; MFI, mean fluorescence intensity; NS, non-stimulated; TGF-β1, transforming growth factor beta 1.

While we previously studied mechanisms of T_reg_ activation in a model of multiple myeloma to therapeutically disengage these cells, here we instead sought to engage the immunoregulatory T_reg_–IFN axis to promote allograft survival. Using flow cytometry (Supplementary Figure S2), we observed that type I IFNs could dose-dependently improve FoxP3^+^ T_reg_ polarization from naïve CD4^+^ T cells in TGF-β1-mediated T_reg_ induction assays, as judged by increased FoxP3 expression (Figure 1A). Additionally, the average proliferative capacity of the conventional CD4 ^+ ^FoxP3^−^ T cells (T_cons_), assessed using the mean fluorescence intensity (MFI) of Ki-67 (Supplementary Figure S3), was robustly diminished in type I IFN-treated T cells, while the Ki-67 MFI in IFN- treated CD4 ^+ ^FoxP3^+^ T_regs_ was akin to that of untreated cells (Figure 1B). Intriguingly, the induction of FoxP3^+^ T_regs_ was not restricted to the IFN-β-treated conditions, as IFN-α treated conditions similarly demonstrated a significant upregulation of FoxP3 expression.

### Type I IFN enhancement of FoxP3 induction does not translate to an allogeneic skin transplant model

**Figure 2:**
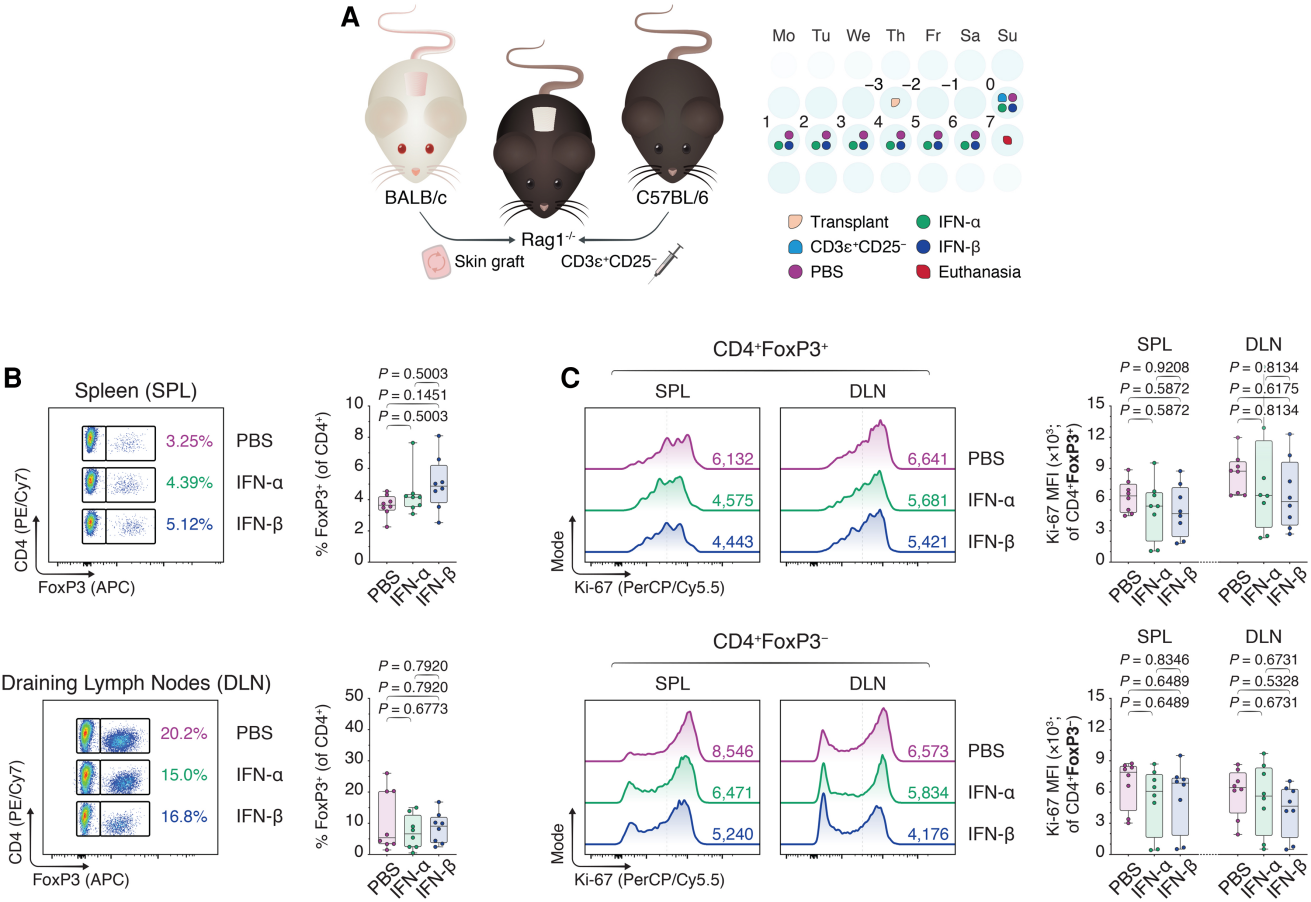
*Type I interferons do not recapitulate enhancement of FoxP3 T_reg_ induction in an allogeneic skin transplant model*. (**A**) Rag1^−/−^ mice were transplanted with BALB/c donor skin three days prior to the adoptive transfer of 1.0 × 10^6^ CD3ɛ^+^CD25^−^ T cells. Afterward, the Rag1^−/−^ mice received daily intraperitoneal injections of 1 × DPBS (PBS), 10,000 IU IFN-α (IFN-α), or 10,000 IU IFN-β (IFN-β) for one week. Seven days from the start of the treatment, the mice were euthanized and the splenocytes together with the draining axillary and brachial lymph nodes were harvested (*n* = 8 biological replicates/condition; 3 experiments). (**B**) Flow cytometric analysis and box plots of FoxP3^+^ T_regs_ among CD4^+^ T cells in the spleens (SPL) and draining lymph nodes (axillary and brachial; DLN). (**C**) Flow cytometric analysis and box plots of Ki-67 MFIs among CD4 ^+ ^FoxP3^+^ T_regs_ and CD4 ^+ ^FoxP3^−^ non-T_regs_. Data represent boxplots with median, interquartile range, minimum, maximum, and all individual data points of the denoted experimental groups. *P* values were calculated with one-way analyses of variance followed by Holm-Šídák multiple comparison tests. DLN, draining lymph nodes; IFN-α, interferon alpha; IFN-β, interferon beta; IL-2, interleukin 2; MFI, mean fluorescence intensity; SPL, spleen; TGF-β1, transforming growth factor beta 1.

To assess if the *in vitro* findings could be extended in an *in vivo* environment, we subsequently grafted BALB/c skin allografts onto the dorsal trunks of immunodeficient Rag1^−/−^ mice on a C57BL/6 background (Figure 2A). Three days later, we intravenously injected 1 × 10^6^ CD3ɛ^+^CD25^−^ C57BL/6 T cells to drive the rejection process and treated the mice once daily with intraperitoneal injections of phosphate-buffered saline (PBS), IFN-α (IFN-α), or IFN-β (IFN-β) suspended in PBS. Seven days following the adoptive transfer, the mice were euthanized and the spleens (SPL) and draining lymph nodes (DLN; axillary and brachial) were harvested (Figure 4A). Using flow cytometry (Supplementary Figure S4), we were unable to observe an increase in CD4 ^+ ^FoxP3^+^ T_regs_ in either the splenic compartment or the DLNs in the IFN-treated conditions (Figure 2B). Additionally, although IFN-α and IFN-β treatment diminished the Ki-67 MFI among CD4 ^+ ^FoxP3^−^ T_cons_ in both draining sites, this difference was statistically insignificant (Figure 2C). Thus, while our *in vitro* findings showed a positive impact of IFN-α and -β on FoxP3 T_reg_ induction, we were faced with additional nuances in the *in vivo* skin transplant setting that prevented us from recapitulating our *in vitro* findings.

### Type I IFNs promote FoxP3 T_reg_ commitment independently of the CD4 population

**Figure 3:**
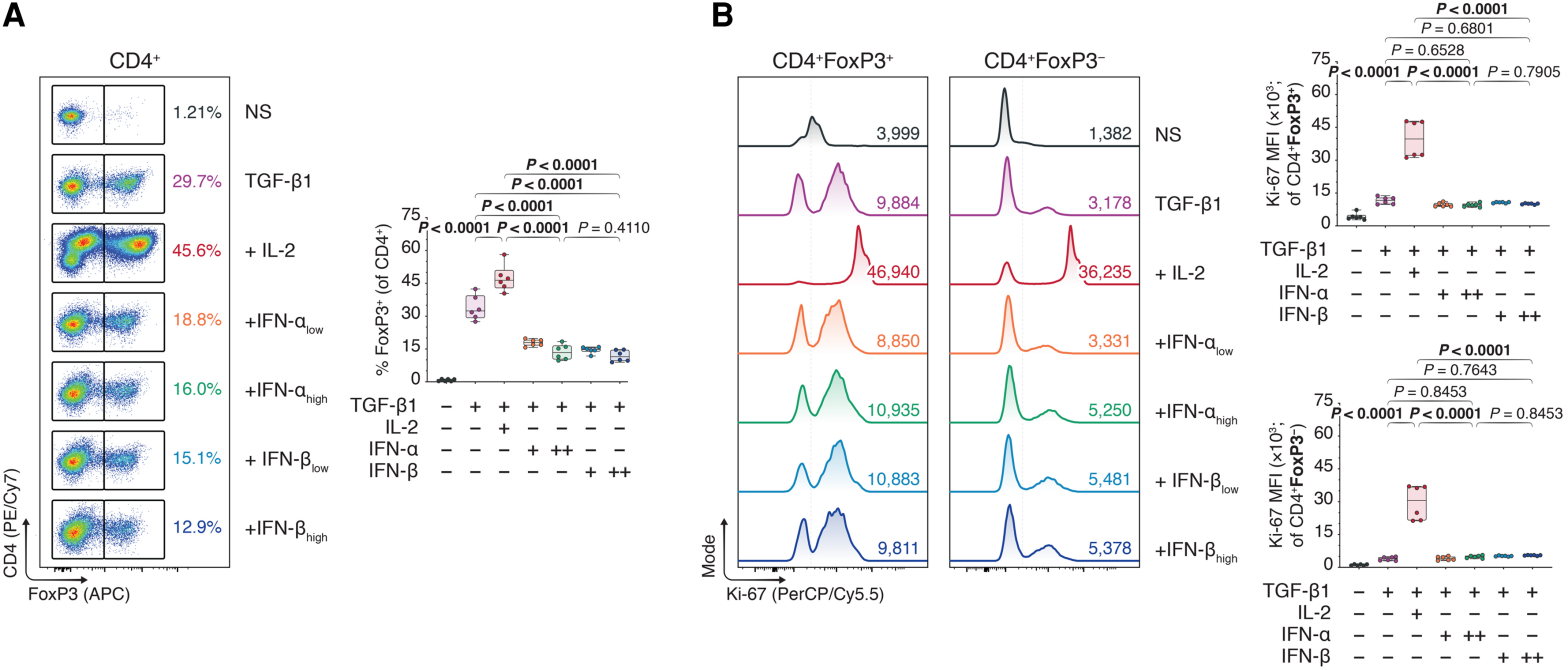
*Type I interferons do not promote FoxP3 T_reg_ inducing effect under strict circumstances of CD4 purity*. Murine CD4 ^+ ^CD25^−^ T cells were magnetically isolated using a double pass CD4 isolation and expanded for three days without stimulants (NS), or with anti-CD3 (1 μg/ml), anti-CD28 (1 μg/ml), and TGF-β1 (10 ng/ml; TGF-β1). In separate wells, IL-2 (20 ng/ml; +IL-2), IFN-α_low_ (1,000 IU/ml; +IFN-α_low_), IFN-α_high_ (10,000 IU/ml; IFN-α_high_), IFN-β_low_ (1,000 IU/ml; +IFN-β_low_), or IFN-β_high_ (10,000 IU/ml; IFN-β_high_) were added to the TGF-β1 condition (*n* = 3 technical replicates/condition; 2 experiments). (**A**) Flow cytometric analysis and box plots of FoxP3^+^ T_regs_ among CD4^+^ T cells. (**B**) Flow cytometric analysis and box plots of Ki-67 mean fluorescence intensities (MFIs) among CD4 ^+ ^FoxP3^+^ T_regs_ and CD4 ^+ ^FoxP3^−^ non-T_regs_. Data represent boxplots with median, interquartile range, minimum, maximum, and all individual data points of the denoted experimental groups. *P* values were calculated with one-way analyses of variance followed by Holm-Šídák multiple comparison tests. IFN-α, interferon alpha; IFN-β, interferon beta; IL-2, interleukin 2; MFI, mean fluorescence intensity; NS, non-stimulated; TGF-β1, transforming growth factor beta 1.

Interestingly, reassessing our former T_reg_ polarization experiments (Figure 1, Supplementary Figure S2), we concluded our CD4^+^ T cell purities (∼60%) were much lower than regularly expected from magnetic-activated cell sorting. We hypothesized the lower CD4^+^ purity—including ∼40% lymphocytes of pan-splenic origin caused by a faulty lot of isolation reagents—was perhaps muddling the induction of FoxP3^+^ T_regs_ upon type I IFN treatment. We speculated that type I IFNs acted directly on the CD4^+^ T cells, and that a purer CD4 T cell population could ameliorate the IFN-dependent treatment outcomes. Performing a double pass magnetic CD4^+^ T cell isolation, we cultured ∼95% pure CD4^+^ T cells (Supplementary Figure S5) in TGF-β1-dependent T_reg_ polarization assays together with IL-2 or different doses of IFN-α and -β. Contrary to our expectations, however, neither IFN-α nor -β elevated FoxP3^+^ induction in the pure CD4^+^ T_cons_ relative to conditions with TGF-β1 alone or TGF-β1 plus IL-2—in fact, even decreasing the FoxP3 expression (Figures 3A and B).

### Type I IFNs augment FoxP3 T_reg_ polarization in concert with ancillary cytokine signals including IL-2

**Figure 4:**
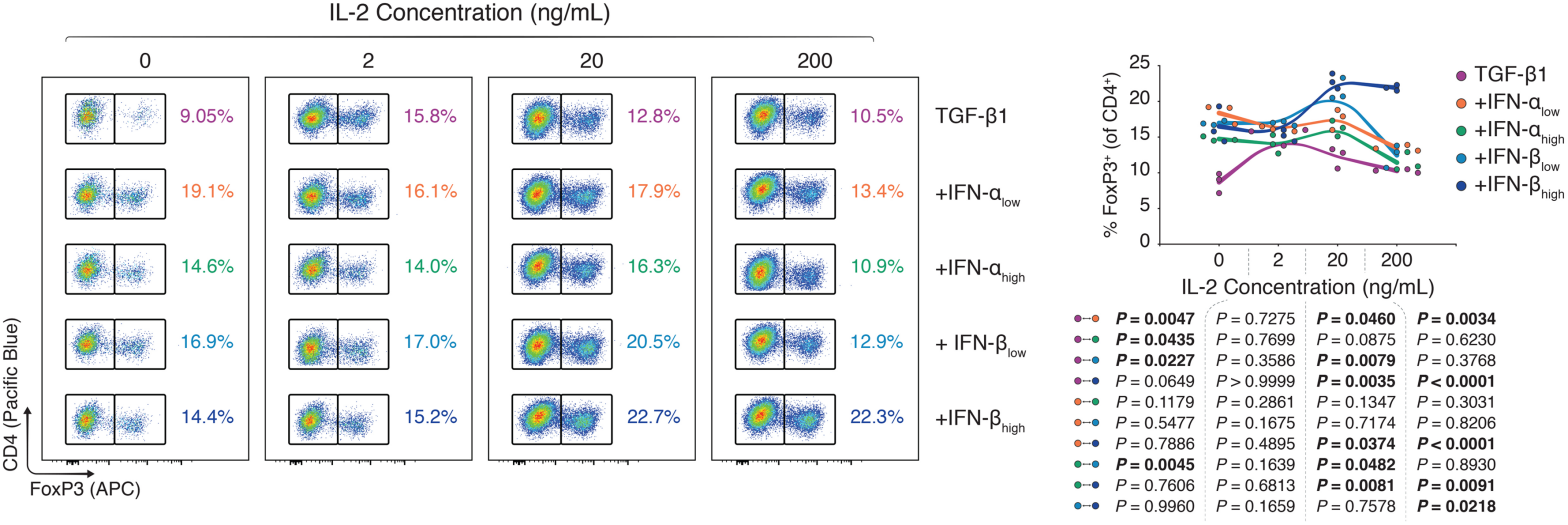
*Type I interferons augment FoxP3 T_reg_ polarization in concert with ancillary cytokine signals*. Murine splenocytes were harvested and expanded for three days with anti-CD3 (1 μg/ml), anti-CD28 (1 μg/ml), and TGF-β1 (10 ng/ml; TGF-β1). In separate wells, IFN-α_low_ (1,000 IU/ml; +IFN-α_low_), IFN-α_high_ (10,000 IU/ml; IFN-α_high_), IFN-β_low_ (1,000 IU/ml; +IFN-β_low_), or IFN-β_high_ (10,000 IU/ml; IFN-β_high_) were added to the TGF-β1 condition. For all three conditions, the IL-2 concentration was titrated (0–200 ng/ml; *n* = 3 technical replicates/condition; 1 of 2 experiments). Flow cytometric analysis and XY plots of FoxP3^+^ T_regs_ among CD4^+^ T cells under varying concentrations of interleukin 2. Data represent all individual data points of the denoted experimental groups. *P* values were calculated with mixed-effects model analyses using the Geisser-Greenhouse correction followed by Tukey multiple comparison tests. IFN-α, interferon alpha; IFN-β, interferon beta; IL-2, interleukin 2; MFI, mean fluorescence intensity; TGF-β1, transforming growth factor beta 1.

Serendipitously, our flawed isolation reagents seemed to have exposed a T-cell-extrinsic factor that was enhancing FoxP3 induction following type I IFN treatment. Synthesizing the observations that (i) impure CD4^+^ T cells experienced greater type I IFN-dependent FoxP3^+^ T_reg_ induction, (ii) sole type I IFN treatment without TGF-β1 precluded FoxP3 induction, and (iii) that type I IFNs were potentially accentuating ancillary cytokines such as IL-2 in our *in vitro* experiments, we performed TGF-β1-dependent T_reg_ polarization assays using whole splenocytes rather than pure CD4^+^ T cells, with different doses of type I IFNs and titrated concentrations of IL-2. In our hands, murine splenocytes are predominantly compound of CD4^+^ T cells (∼30%), CD8α^+^ T cells (∼15%), and B220^+^ B cells (∼40%; Supplementary Figure S6). Whole splenocytes, notably, include a broad array of leukocytes, ranging from T cells to B cells, monocytes, macrophages, dendritic cells, granulocytes, and natural killer cells (10). Using flow cytometry (Supplementary Figure S7) we observed that type I IFNs dose-dependently boosted FoxP3^+^ T_reg_ induction in the splenocyte cultures, however, co-dependently on the IL-2 concentration. While 2 ng/ml of IL-2 yielded no difference in the FoxP3 induction among type I IFN-treated and -untreated splenocytes, at 20 and 200 ng/ml of IL-2, there were marked differences in the CD4 ^+ ^FoxP3^+^ frequency between TGF-β1-treated splenocytes with and without type I IFNs (Figure 4).

## Discussion

Despite a growing corpus of evidence supporting the immunomodulatory properties of type I IFNs, the literature is conflicted on the role of type I IFNs and their regulation of T-cell responses in varying disease models. Though it is undeniable that type I IFNs are involved in cellular processes ranging from viral immunity to vascular remodeling and immunoregulation of adaptive and innate immune cells (2–4), the circumstances under which these IFNs are immunostimulatory or -suppressive, and how the type I IFN treatment can be steered in shaping immune responses remains poorly defined. Nonetheless, over the years, clinicians have explored the use of type I IFNs in disparate pathologies with varying degrees of success, ranging from hematological malignancies (chronic myelogenous leukemia, multiple myeloma, non-Hodgkin lymphoma), to viral diseases (chronic hepatitis B and C, cytomegalovirus, human papillomavirus), and autoimmunity (most notably, multiple sclerosis) (6, 11). For relapsing-remitting multiple sclerosis specifically, IFN-β treatment has been a first-line, disease-modifying therapy for over two decades due to its long-term safety profile and therapeutic efficacy (12–14).

Our group's foray into type I IFN biology began with the finding that patients with more severe, fast-progressive multiple myeloma experienced heightened type I IFN signaling and concomitantly showcased upregulated T_reg_ responses (4). Using animal models of multiple myeloma, we found that both the depletion of all FoxP3^+^ T_regs_ and the selective ablation of IFNAR1 on T_regs_ induced tumor remission in an aggressive murine myeloma model (4). To take this understanding and develop it further in the transplant domain, where the opposite effect is desired—that is, T_reg_ engagement rather than disengagement—we began exploring the feasibility of exploiting type I IFNs for therapeutic purposes following transplantation. Here, using TGF-β1-dependent T_reg_ induction assays, we found that both IFN-α and -β could enhance the conversion of naïve CD4^+^ T cells into FoxP3^+^ T_regs_. Notably, we observed that this elevated commitment of CD4^+^ T cells to the T_reg_ lineage was co-dependent on ancillary cytokine signals, including IL-2, rather than independently arising from type I IFNs signals alone.

Interestingly, the role of type I IFNs in transplantation was also recently described by fellow transplant researchers, providing evidence of IFN-β-directed pathways augmenting FoxP3^+^ T_reg_ induction and prolonging allograft survival in a heterotopic heart transplantation model (7). The T_reg_ dependence of these IFN-β-specific treatment outcomes was underscored with *in vivo* depletion experiments of adoptively-transferred FoxP3^+^ T_regs_ from FoxP3 diphtheria toxin receptor (FoxP3^DTR^) mice causing accelerated rejection of allogeneic heart transplants (7). The authors of this study proposed a minimalistic, direct model of IFN-β-mediated FoxP3 induction, excluding other type I IFN signals such as IFN-α. Though it is tempting and sometimes necessary to formulate reductionist immunological theories, however, we believe the selective effect of IFN-β on T_reg_ induction to be more nuanced, not to exclude IFN-α signals, and to be orchestrated by a complex set of ancillary cytokine signals, at least based on our findings presented here.

In our experiments we assessed the role of both IFN-α and -β in steering FoxP3^+^ Treg commitment, where IFN-α is characterized as having lower affinity engagement to IFNAR than IFN-β. Notably, we observed that IFN-α (specifically isoform 2) could statistically significantly increase FoxP3^+^ T_reg_ induction, to a degree similar to IFN-β albeit somewhat less potently. In a recent publication by Vitale and colleagues, the therapeutic use of IFN-α (isoform 4) was similarly explored, but instead exploring *in vitro* and *in vivo* models of antigenic tolerance induction and colitis (15). Corroborating with our *in vitro* findings, the authors described a synergy between IFN-α and TGF-β1 with IL-2 in the induction, maintenance, and differentiation of FoxP3^+^ T_regs_. Moreover, they also found that both IFN-α and IL-2 signals were required for naïve CD4^+^ T cells to acquire the FoxP3^+^ T_reg_ phenotype—IFN-α sustaining but not replacing the IL-2 signals (15). It is worth noting that Vitale and colleagues did not compare IFN-α and IFN-β head-to-head in their *in vivo* experiments and did not perform transplant models. Still, their work and ours support a non-negligible role for IFN-α in the regulation of *in vitro* and *in vivo* T_reg_ responses, in contrast to the findings described by Fueyo-González and colleagues (7).

Looking at the available body of literature, it is certainly likely that IFN-α has a similar biological effect as IFN-β since both signal through the same IFNAR receptor, the only difference being their relative affinities for IFNAR (3). Once threshold cytokine concentrations for engagement are surpassed, IFN-α and -β should provide the same level of IFNAR signaling and enact comparable, if not indistinguishable, downstream effects. In other words, if the relative signaling strengths of IFN-α and -β and their cognate receptors can be leveled—by accepting higher absolute concentrations of IFN-α than IFN-β—the therapeutic outcomes should hypothetically be the same.

Beyond questions of biochemical and signaling similarities between the different type I IFNs, there is an innate pleiotropy to type I IFN signals downstream of IFNAR, possibly making it too ambitious to derive a simple yet comprehensive model that predicts outcomes of type I IFN treatment across tissues, cell types, and cytokine milieus (3). Importantly, a discrete number of type I IFN inputs can be fine-tuned into a broad range of signaling outputs by spatiotemporally-dictated factors at the macroscopic (cytokine milieu, cell type) and microscopic level (DNA transcription, mRNA translation, epigenetic repression) to sculpt immune responses most appropriate for host defenses and cell survival (16). Canonically, type I IFNs activate Janus kinase 1 (Jak1) and tyrosine kinase 2 (Tyk2) downstream of IFNAR, phosphorylating heterodimers of STAT1/STAT2 followed by linkage of pSTAT1/-2 with IFN regulatory factor 9 (IRF9) and formation of the IFN-stimulated gene factor 3 (ISGF3) complex—thus, triggering over 1,000 ISGs involved in a myriad of cellular processes (3, 17). Nonetheless, type I IFNs can effectively activate all seven known STATs and propagate signals through extracellular signal-regulated kinase (ERK), p38 mitogen-activated protein kinase (MAPK), phosphoinositide 3-kinase (PI3K), and protein kinase C (PKC) pathways (3, 17). Additionally, type I IFNs can augment auxiliary cytokine responses, intersecting with unrelated cytokine pathways such as IFN-γ and IL-6 (5), and facilitate STAT-mediated epigenetic modifications such as chromatin remodeling *via* histone acetylation (1, 16).

Thus, the fascinating complexity of type I IFN signaling makes modeling of its therapeutic outcomes understandably intricate, as different cell types activate disparate STATs in response to the same IFN (6), acute vs. chronic signaling respectively shifts its effects from being immunostimulatory to -regulatory (16), and priming with type I IFNs prior to an antigenic stimulus dictates the exposure's stimulatory or suppressive nature (17). These very nuances can, in part, explain the findings by Fueyo-González and colleagues (7), as type I IFNs can promote dendritic cell (DC) maturation (18) to subvert paradoxical allo-immunity through immunogenic DCs. Additionally, type I IFNs can elevate IL-2 sensitivity in T cells (18), with IFNs plausibly magnifying FoxP3^+^ induction in concert with IL-2. Intriguingly, early *in vitro* exposure to IFN-β yielded maximal STAT1-dependent FoxP3^+^ induction in their hands—the IFN-β-dependent effects rapidly dwindling after antigenic stimulation—while the IFN-β-dependent heart transplant survival relied on early IFN-β treatment initiated one day prior to the transplantation (7).

The importance of the timing of IFN signal relative to other immunological signals is an intriguing concept. Previously, it has been posited that T-cell activation redraws the transcriptomic landscape to restrain the transcription response to IFNs. T cells are, correspondingly, characterized by a dynamic stoichiometry of STATs that compete for downstream effector proteins (5). When T-cell receptor (TCR) stimulation coincides with, or shortly precedes, IFNAR signaling, type I IFNs induce STAT4-dependent cell survival, proliferation, and differentiation, while in cases that IFNAR signaling precedes TCR engagement, the STAT1-dependent anti-proliferative and pro-apoptotic capacity prevails (18). Indeed, in the survival experiments with or without type I IFN following heterotopic heart transplantation (7), FoxP3^+^ T_reg_ induction was primarily driven by STAT1 activation with smaller contributions by STAT4 and STAT5, again, as IFN-β treatment was started the day before the transplant—thus, priming the immune compartments with IFN-β prior to TCR signaling.

We consider the very complexity of type I IFN signaling to explain our findings described here, where FoxP3^+^ T_reg_ responses could be enhanced *in vitro* with cultures of impure CD4^+^ T cells and whole splenocytes, but not with pure CD4^+^ and CD8^+^ T cells in the more multifaceted *in vivo* skin transplant model. By looking at our phenotyping of the splenocyte compartment, in which ∼90% of the cells were either CD4^+^ T cells, CD8^+^ T cells, or B220^+^ B cells, by sheer quantity alone it would seem reasonable to assume the type-I-IFN-mediated enhancement of FoxP3^+^ T_reg_ induction would be modulated by either T or B cells. Nonetheless, there is evidence of a much greater microcosm of lymphocytic cellular constituents in the murine spleen, including natural killer cells, immature myeloid cells, neutrophils, macrophages, myeloid dendritic cells, and plasmacytoid dendritic (10) that likely play a key role in determining type-I-IFN-dependent outcomes. Among these, the antigen-presenting cells (APCs) in the form of DCs are intriguing candidates for further assessment for type-I-IFN-direct FoxP3^+^ T_regs_ effects. Notably, DCs are known to induce tolerance in sites of antigen encounter both centrally and in the periphery (19), being especially important in maintaining immune homeostasis and blocking autoimmune responses in settings of peripherally-induced T-cell tolerance (20). Overall, we believe, our findings described here constitute seed knowledge for future explorations towards the cellular constituents that determine enhanced FoxP3^+^ T_reg_ responses *via* type I IFN signals. These cellular constituent will have to excavated using multidisciplinary approaches including mouse models with dysregulated type I IFN responses in selected lymphoid subsets—perhaps even of the endothelial cell populations in the lymphoid stroma—to unearth the cells responsible for arbitrating type-I-IFN-specific FoxP3^+^ T_reg_ responses.

In terms of human translatability, future research efforts will have to determine if the tolerogenic effects exacted by type I IFNs can be reproduced in human FoxP3^+^ T_regs_ and be exploited for organ transplantation. Importantly, such enhanced understanding of type I IFN signaling in human immunology can permit clinicians to diversify and strengthen the available immunosuppressive therapy options. Finding alternatives to traditional immunosuppressants and broadening the available immunosuppressive arsenal can bolster clinical efforts in steering clear of the marked side-effects associated with the currently available standard-of-care drugs, such as calcineurin inhibitors including tacrolimus, and approach operational tolerance (21) to human organ allografts.

Taken together, considering the promising nature of therapeutically manipulating the T_reg_-IFN signaling axis, garnering insights on the factors that sculpt the transcriptomic landscape after type I IFN treatment; including chronic treatment regiments, longer priming, and co-dependent cytokine signals; could permit maximal exploitation of therapeutic strategies that have clinical translation potential.

### Limitations

One limitation of all T_reg_ studies—murine and human alike—is the methodological constraint of attaining ample T_regs_ for experimental studies due to the number of available T_regs_ per murine spleen. Specifically, every euthanized mouse provides only one million T_regs_. Achieving enough T_regs_ for *in vitro* and *in vivo* experiments with a multitude of treatment conditions is thus extremely challenging. Future research towards alternative *in vitro* and *in vivo* experimental T_reg_ models is warranted to increase the number of testable conditions and allow a more in-depth assessment of type-I-IFN-driven signaling in stabilizing the FoxP3^+^ T_reg_ phenotype. Indeed, to aid the potential clinical translation of type I IFN therapy in the context of tissue and organ transplantation, more insights need to be gathered on the spatiotemporal particularities of type I IFNs in dictating their therapeutic outcomes. In the future, we hope to optimize models that will allow us to pick apart these particularities. Identifying exact dosages and temporal circumstances under which type I IFNs dependently provide immunosuppressive effects will be essential to expand on the results presented here.

## Conclusion

Accruing evidence supports type I IFN-dependent promotion of T_reg_ polarization from naïve helper CD4^+^ T cells in various transplant settings. The IFN-dependent effects herein likely rely on suppressing paradoxical immunogenicity and priming of the host with type I IFN in concert with ancillary cytokine signals. Eyeing clinical translation of type I IFN therapies for transplantation, future studies that extricate the factors that sculpt the immunological responses to type I IFN treatment are warranted.

## Data Availability

'The original contributions presented in the study are included in the article/**Supplementary Materials**, further inquiries can be directed to the corresponding authors.
