# Supplementary material for: Type I interferons augment regulatory T cell polarization in concert with ancillary cytokine signals
Source: Front Transplant. 2023 Apr 17;2:1149334. doi: 10.3389/frtra.2023.1149334 (PMC11235373; doi:10.3389/frtra.2023.1149334)
Supplement: Supplementary file 2 [file Presentation1.pdf]

Type I interferons augment regulatory T cell polarization in concert with ancillary cytokine signals

Siawosh K. Eskandari[1,2*], Hazim Allos[1], Jenelle M. Safadi[1,3], Ina Sulkaj[1,4], Jan S.F. Sanders[2], Paolo Cravedi[5], Irene M. Ghobrial[6], Stefan P. Berger[2], Jamil R. Azzi[1*]

[1] Transplantation Research Center, Division of Nephrology, Brigham and Women's Hospital, Harvard Medical School, Boston, MA, United States.

[2] Division of Nephrology, University Medical Center Groningen, University of Groningen, Groningen, the Netherlands.

[3] Perelman School of Medicine, University of Pennsylvania, PA, United States.

[4] Graduate Program in Immunology, Johns Hopkins School of Medicine, Baltimore, MD, United States.

[5] Translational Transplant Research Center, Division of Nephrology, Icahn School of Medicine at Mount Sinai, New York City, NY, United States.

[6] Department of Medical Oncology, Dana-Farber Cancer Institute, Harvard Medical School, Boston, MA, United States.

***Correspondence:**
Siawosh K. Eskandari (s.eskandari@umcg.nl);
Jamil R. Azzi (jazzi@bwh.harvard.edu).

Supplemental Materials

SUPPLEMENTARY METHODS

T cell isolation

For in vitro analyses of FoxP3^{+} T_{reg} induction, CD4^{+}CD25^{-} T cells were isolated from murine spleens using a two-step MACS-based approach as per the manufacturer's protocol (#130-091-041, Miltenyi Biotec). To attain a higher CD4^{+} T cell purity in highlighted experiments, a double LD column pass was performed. For the adoptive transfer of CD3ε^{+}CD25^{-} T cells, CD3ε^{+} T cells were first isolated from murine spleens using a MACS-based negative selection kit (#130-096-535, Miltenyi Biotec) followed by the depletion of CD25^{+} T cells using a positive selection kit (#130-091-072, Miltenyi Biotec), both as per the manufacturer's protocol.

Skin allotransplantation model

Full-thickness trunk skin grafts (1.0 cm × 1.5 cm) from BALB/c donors were harvested at the level of the areolar connective tissue, and, connective, adipose, and panniculus carnosus tissues were cleared using blunt-tipped forceps. The fur of each anesthetized recipient Rag1$^{-/-}$ mouse was shaven at the dorsal trunk, 1.0–by–1.5 cm of the recipient's skin was excised, and an equally-sized skin graft was sutured onto the graft bed with four single sutures using PERMA-HAND 4-0 Silk Suture (#1677G, Ethicon). Skin transplants were secured with dry gauze and bandaged for seven days.

T cell harvesting

To isolate lymphocytes from the skin-transplanted mice, the mice were euthanized and the spleens (SPL) and axillary and brachial lymph nodes (draining lymph nodes; DLN) were harvested. The spleens and DLN were then mechanically dissociated and homogenized into single-cell suspensions under sterile conditions and were filtered through 70-μm cell strainers. Homogenized cell suspensions were used for downstream flow cytometry analysis.

Flow cytometry

Analysis of lymphocytes for in vitro and in vivo studies was performed with fluorochrome-conjugated monoclonal antibodies against both surface-expressed and intracellular epitopes. First, cells were prepared for flow cytometry by staining surface epitopes for 25 min at 4°C in flow staining buffer, consisting of 1× DPBS supplemented with 1.0% (w/v) bovine serum albumin

(#A2153, Sigma-Aldrich) and 0.020% sodium azide (#S8032, Sigma-Aldrich). Afterward, cells were fixed and permeabilized with the eBioscience FoxP3 Fixation/Permeabilization concentrate and diluent cocktail (#00-5523-00, Invitrogen) for 30 min at 4°C. Finally, intracellular epitopes were stained in 1× permeabilization buffer diluted from 10× eBioscience FoxP3 Permeabilization Buffer (#00-5523-00, Invitrogen) with deionized water. Stained cells were analyzed on a FACS Canto II (BD Biosciences) flow cytometer, and the resultant flow cytometry standard (FCS) files were analyzed with FlowJo version 10 (FlowJo LLC).

Below, the fluorochrome-conjugated antibodies used in described experiments have alphanumerically been listed.

Table S1. List of the flow cytometry antibodies.

Antibody	Reactivity	Fluorophore	Clone	Dilution	Vendor	Catalogue ID
Annexin V	Universal	BV510	N/A	1:125 µL	BioLegend	640937
Anti-CD4	Mouse	PE/Cy7	RM4-5	1:500 µL	BioLegend	100528
Anti-CD8α	Mouse	APC	53-6.7	1:500 µL	BioLegend	100711
Anti-CD45R/B220	Mouse, Human	PerCP/Cy5.5	RA3-6B2	1:500 µL	BioLegend	103233
Anti-CD69	Mouse	PE/Cy7	H1.2F3	1:500 µL	BioLegend	104511
Anti-FoxP3	Mouse	APC	FJK-16s	1:125 µL	Invitrogen	17-5773-82
Anti-Ki-67	Mouse	PerCP/Cy5.5	16A8	1:250 µL	BioLegend	652423
Fixable Viability Dye	Universal	eFluor 450	N/A	1:1000 µL	eBioscience	65-0863-14
Fixable Viability Dye	Universal	eFluor 780	N/A	1:1000 µL	eBioscience	65-0865-18

SUPPLEMENTARY FIGURES

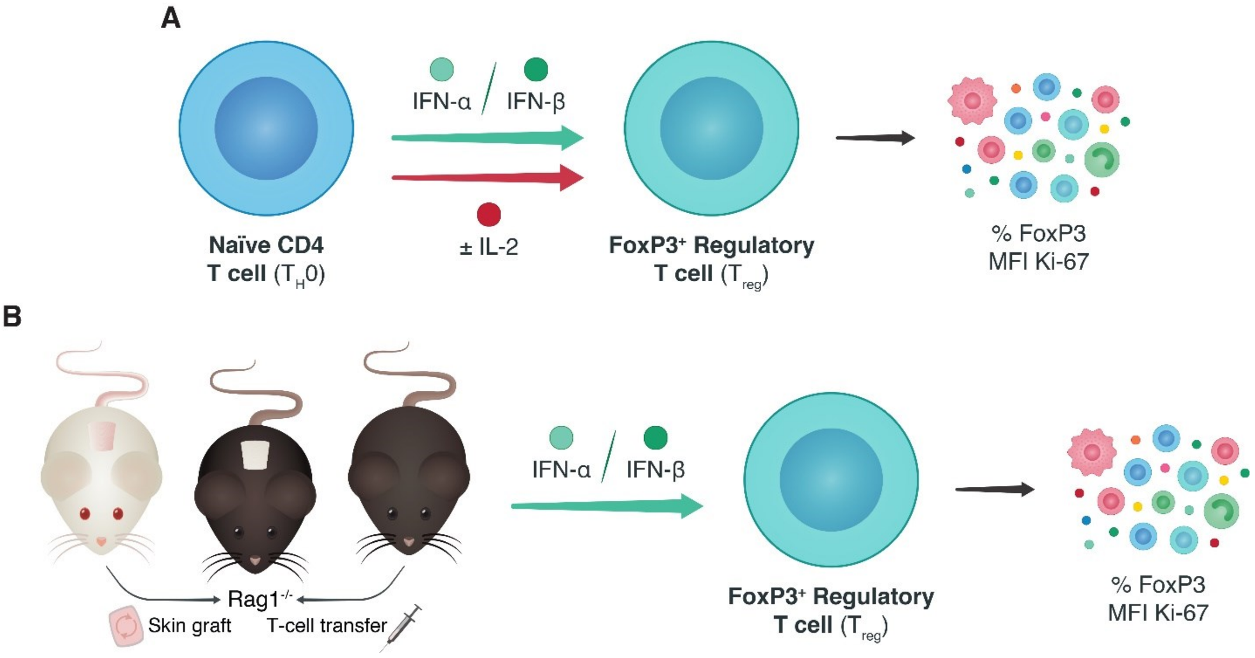

FIGURE S1 | *Study design investigating the impact of type I interferons on augmenting $CD4^{+}FoxP3^{+}$ regulatory T cell induction.* (**A**) We studied the type I interferon (IFN-)directed modulation of $FoxP3^{+}$ T_{regs} homeostasis as well as T_{reg} activation in the presence of IFN-α and IFN-β signals using in vitro T_{reg} induction assay with or without IL-2, followed by flow cytometric assessment of the FoxP3 percentage (%) and the Ki-67 mean fluorescence intensity (MFI). (**B**) Building on the in vitro experiments, we next sought to assess the effects of IFN-α and IFN-β in the context of an allogeneic skin transplant model using $Rag1^{-/-}$ mice as transplant recipients, followed by flow cytometric assessmen of the FoxP3 % and the Ki-67 MFI in the spleens and draining lymph nodes of transplanted and treated mice. IFN-α, interferon alpha; IFN-β, interferon beta; IL-2, interleukin 2; MFI, mean fluorescence intensity.

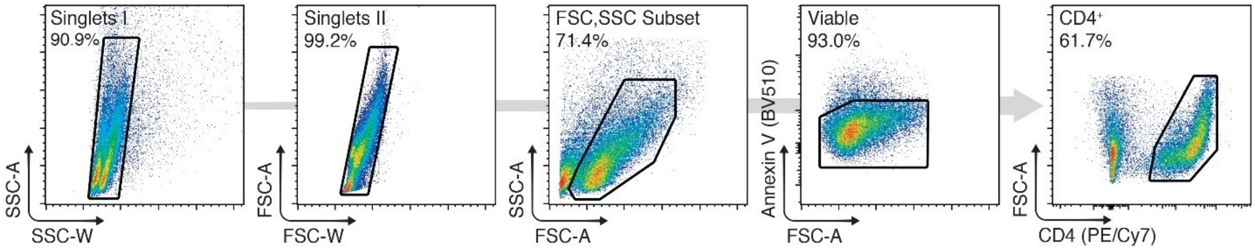

FIGURE S2 | *Gating strategy identifying in vitro cultured murine CD4⁺ T cells in Figure 1.*

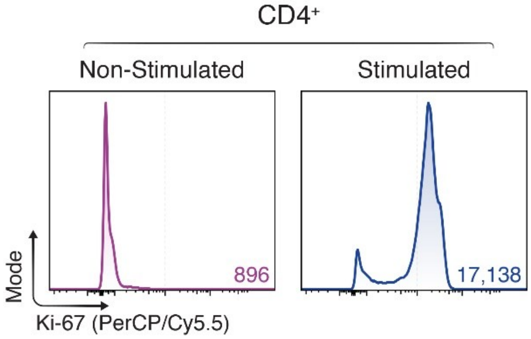

FIGURE S3 | *Representative gating of Ki-67 expression among unstimulated and three-day stimulated CD4⁺ T cells.* Murine CD4⁺CD25⁻ T cells were magnetically isolated as per the manufacturer's instructions and expanded for three days without stimulants (unstimulated), or with 1 μg/mL anti-CD3, 1 μg/mL anti-CD28, 10 ng/mL TGF-β1, and 20 ng/mL IL-2 (stimulated). The numbers in the plots indicate the mean fluorescence intensity of Ki-67 for each plot.

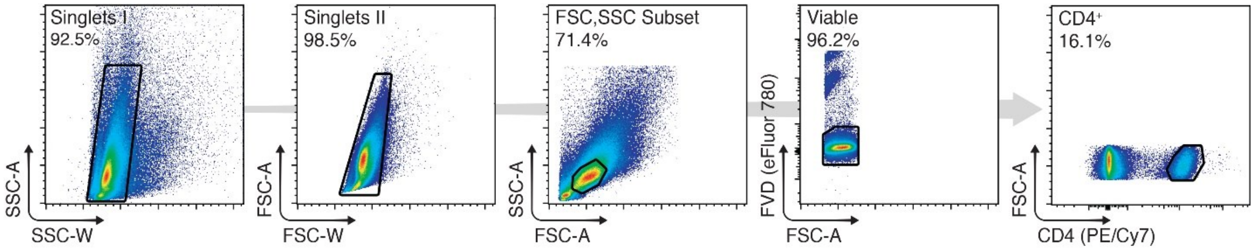

FIGURE S4 | *Gating strategy identifying in vivo derived murine CD4⁺ T cells in Figure 2.* FVD, fixable viability dye.

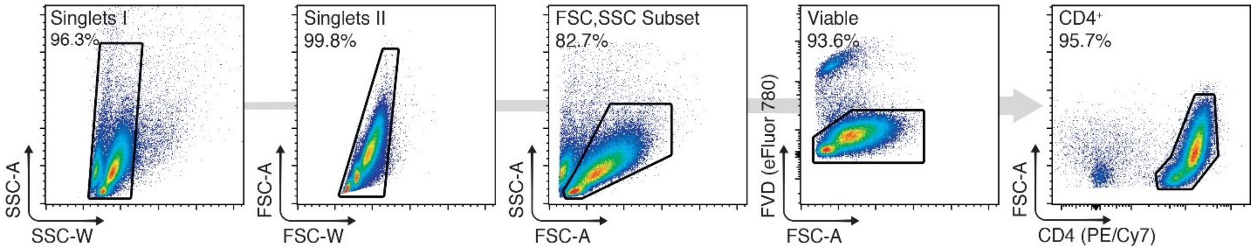

FIGURE S5 | *Gating strategy identifying in vitro cultured murine CD4⁺ T cells in Figure 3.* FVD, fixable viability dye.

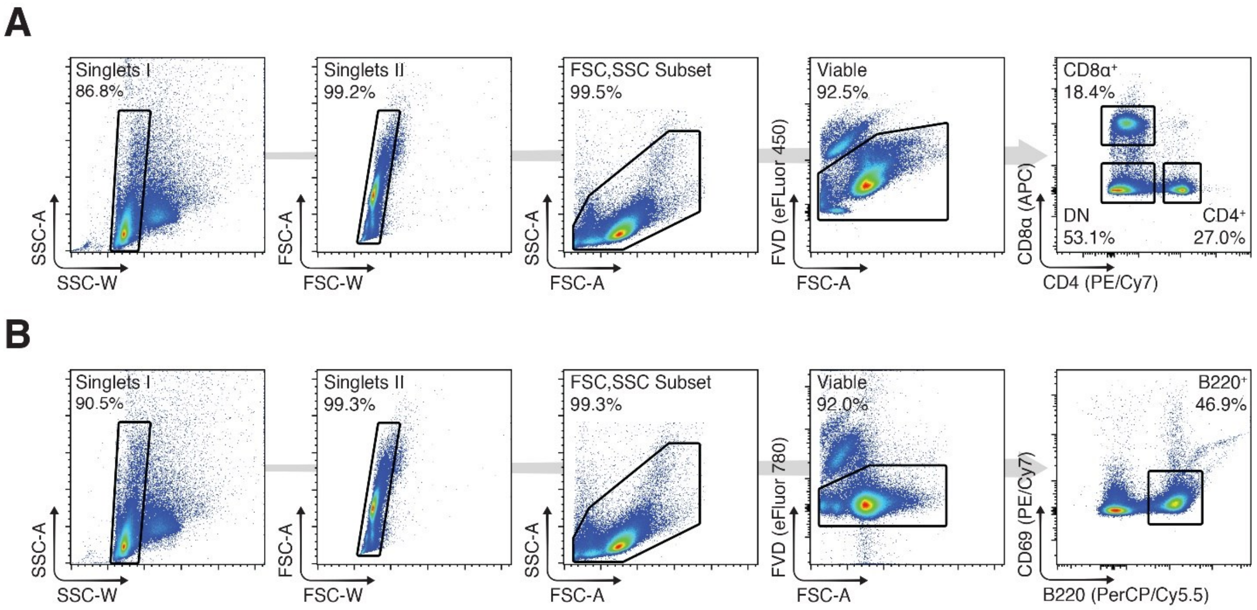

FIGURE S6 | *Representative naïve murine splenocyte compartment characterization.* (**A**) Gating strategy demonstrating ~30% helper CD4⁺ T cells and ~15% cytotoxic CD8α⁺ T cells in the splenic compartment. (**B**) Gating strategy demonstrating ~45% B220⁺ B cells in the splenic compartment. DN, double negative; FVD, fixable viability dye

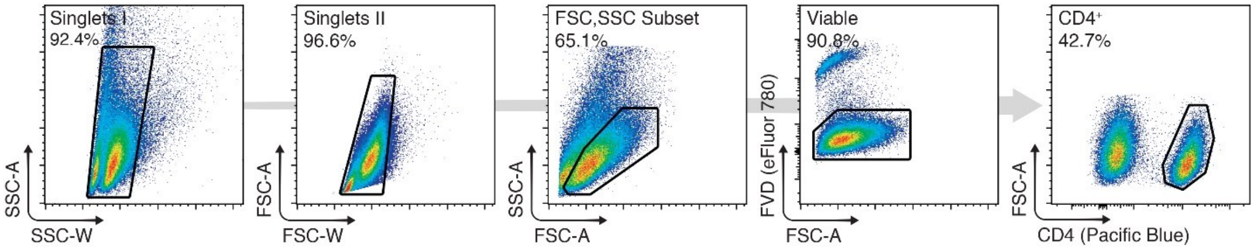

FIGURE S7 | *Gating strategy identifying in vitro cultured murine CD4⁺ T cells in Figure 4.* FVD, fixable viability dye.
